# A nontoxic and low-cost hydrothermal route for synthesis of hierarchical Cu_2_ZnSnS_4_ particles

**DOI:** 10.1186/1556-276X-9-208

**Published:** 2014-05-04

**Authors:** Yu Xia, Zhihong Chen, Zhengguo Zhang, Xiaoming Fang, Guozheng Liang

**Affiliations:** 1 Jiangsu Key Laboratory of Advanced Functional Polymer Design and Application, College of Chemistry, Chemical Engineering, and Materials Science, Soochow University, Suzhou 215123, China; 2 Key Laboratory of Enhanced Heat Transfer and Energy Conservation, Ministry of Education, School of Chemistry and Chemical Engineering, South China University of Technology, Guangzhou 510640, China; 3 Suzhou Jufeng Electrical Insulation System Co. Ltd, Suzhou 215214, China

**Keywords:** Cu_2_ZnSnS_4_, Nanocrystalline material, Hierarchical particles, Hydrothermal process, Photoelectrochemical property

## Abstract

We explore a facile and nontoxic hydrothermal route for synthesis of a Cu_2_ZnSnS_4_ nanocrystalline material by using l-cysteine as the sulfur source and ethylenediaminetetraacetic acid (EDTA) as the complexing agent. The effects of the amount of EDTA, the mole ratio of the three metal ions, and the hydrothermal temperature and time on the phase composition of the obtained product have been systematically investigated. The addition of EDTA and an excessive dose of ZnCl_2_ in the hydrothermal reaction system favor the generation of kesterite Cu_2_ZnSnS_4_. Pure kesterite Cu_2_ZnSnS_4_ has been synthesized at 180°C for 12 h from the reaction system containing 2 mmol of EDTA at 2:2:1 of Cu/Zn/Sn. It is confirmed by Raman spectroscopy that those binary and ternary phases are absent in the kesterite Cu_2_ZnSnS_4_ product. The kesterite Cu_2_ZnSnS_4_ material synthesized by the hydrothermal process consists of flower-like particles with 250 to 400 nm in size. It is revealed that the flower-like particles are assembled from single-crystal Cu_2_ZnSnS_4_ nanoflakes with *ca.* 20 nm in size. The band gap of the Cu_2_ZnSnS_4_ nanocrystalline material is estimated to be 1.55 eV. The films fabricated from the hierarchical Cu_2_ZnSnS_4_ particles exhibit fast photocurrent responses under intermittent visible-light irradiation, implying that they show potentials for use in solar cells and photocatalysis.

## Background

The quaternary Cu_2_ZnSnS_4_ (CZTS) compound, derived from CuInS_2_ by replacing In(III) with Zn(II) and Sn(IV), has the advantages of optimum direct band gap (around 1.5 eV) for use in single-junction solar cells, abundance of the constituent elements, and high absorption coefficient (>10^4^ cm^-1^) [1-5]. Thus, increasing attention has been paid on CZTS materials in recent years [6-10]. Low-cost solar cells based on CZTS films as absorber layers have achieved an increasing conversion efficiency [11-15]. CZTS nanocrystalline materials have been found to show potentials for use in negative electrodes for lithium ion batteries [16] and counter electrodes for high-efficiency dye-sensitized solar cells [17-19] and as novel photocatalysts for hydrogen production [20]. Obviously, an environment-friendly and low-cost synthesis route for the large-scale production of CZTS in high quality is an essential prerequisite for its applications in all those fields.

At present, the routes for synthesis of CZTS nanocrystalline materials can be subsumed under two broad categories: the hot-injection method [12,21-23] and the solvothermal process [13,18,24-26]. Although the hot-injection method can be used to synthesize CZTS nanocrystals with narrow size distribution, this method suffers from several shortcomings such as the need of expensive raw materials with high levels of toxicity, complicated processes, and high reaction temperatures (above 250°C). In contrast with the hot-injection method, the solvothermal process, which usually produces hierarchical CZTS particles by one-pot reaction, possesses the advantages of simple process and relative cheap raw materials. Furthermore, it has been found that hierarchical particles can provide a large surface area along with the functions of generating light scattering and favoring electron transport, as compared with nanocrystals [13]. Up to now, anhydrous ethylenediamine [24,26], the mixture of ethylenediamine and water [27-29], ethylene glycol [13,18], triethylene glycol [18], and dimethyl formamide (DMF) [30] have been used as a solvent for the solvothermal method, respectively. In contrast with those organic solvents, water is much cheaper and more environment-friendly. Undoubtedly, if water is used to replace these organic solvents, a hydrothermal route will be developed, which is more desirable for the environment-friendly and low-cost synthesis of CZTS nanocrystalline materials. However, few investigations on synthesis of CZTS nanocrystalline materials by the hydrothermal method have been reported, except the hydrothermal reactions with Na_2_S [31] or thiourea [32] as the sulfur source. Note that selecting a suitable sulfur source is important for exploring a green hydrothermal process for preparing CZTS nanocrystalline materials. It has been reported that H_2_S is usually generated as a toxic and corrosive intermediate product from the reaction systems containing sulfur, Na_2_S, or thiourea as the sulfur source [33]. Different from those sulfur sources, l-cysteine has been used to prepare metal sulfide nanomaterials without the generation of H_2_S as a by-product [30]. Thus, in the current work, by the aid of ethylenediaminetetraacetic acid (EDTA) as a complexing agent, a low-cost and nontoxic hydrothermal route for synthesis of CZTS has been developed by using water as the solvent and l-cysteine as the sulfur source. The effects of the amount of EDTA, the mole ratio of the three metal ions, and the hydrothermal temperature and time on the phase composition of the obtained samples have been systematically investigated. The phase composition of the obtained CZTS sample has been further confirmed by Raman spectrometry. The microstructure and morphology of the pure CZTS sample have been characterized, and its optical absorption property has been examined. Moreover, the prepared CZTS nanocrystalline material has been employed to fabricate films, and the photoelectrochemical property of the obtained films has been evaluated.

## Methods

### Synthesis of CZTS

CuCl_2_ · 2H_2_O, ZnCl_2_, SnCl_2_ · 2H_2_O, l-cysteine, and EDTA were of analytical grade and used as received without further purification. In a typical synthesis, 2 mmol CuCl_2_ · 2H_2_O, 2 mmol of ZnCl_2_, 1 mmol of SnCl_2_ · 2H_2_O, 4 mmol of l-cysteine, and 0 to 3 mmol of EDTA were dispersed in 20 ml of deionized water for 5 min under constant stirring, and then the obtained solution was transferred to an acid digestion bomb (50 ml). The hydrothermal synthesis was conducted at 170°C to 190°C for 6 to 16 h in an electric oven. After synthesis, the bomb was cooled down naturally to room temperature. The final product was filtrated and washed with 30% and 80% ethanol, followed by drying at 60°C in a vacuum oven. Moreover, in order to investigate the mole ratio of the three metal ions (Cu/Zn/Sn) in the reaction system on the phase composition of the obtained product, three samples were synthesized at 2:1:1, 2:2:1, and 2:3:1 of Cu/Zn/Sn, respectively.

### Characterizations

Powder X-ray diffraction (PXRD) patterns of samples were performed on a Bruker D8 ADVANCE diffraction system (Bruker AXS GmbH, Karlsruhe, Germany) using Cu Kα radiation (*λ* = 1.5406 Å), operated at 40 kV and 40 mA with a step size of 0.02°. The morphology of the pure CZTS sample was observed by using a scanning electron microscope (SEM, Nova Nano 430, FEI, Holland). Transmission electron microscopy (TEM) and high-resolution transmission electron microscopy (HRTEM) images were obtained by using a JEOL JEM-2100 F field emission electron microscope (JEOL Ltd., Akishima, Tokyo, Japan). The Raman spectrum of the sample was recorded on a microscopic Raman spectrometer (LabRAM Aramis, Horiba Jobin Yvon Inc., Edison, NJ, USA). The diffuse reflectance spectrum (DRS) of the CZTS sample was obtained by using a Shimadzu U-3010 spectrophotometer (Shimadzu Corporation, Nakagyo-ku, Kyoto, Japan) equipped with an integrating sphere assembly.

### Photoelectrochemical measurement

The prepared CZTS sample was used to fabricate films as follows: 0.05 g of the sample was mixed with ethanol followed by ultrasound. The obtained CZTS ‘ink’ was then coated onto indium-tin (ITO) oxide glass by spin coating for several times, followed by drying at 120°C for 1 h. Photoelectrochemical measurements were conducted on the obtained CZTS films. Photocurrents were measured on an electrochemical analyzer (CorrTest CS350, CorrTest Instrument Co., Wuhan, China) in a standard three-electrode system by using the prepared CZTS film as the working electrode, a Pt flake as the counter electrode, and Ag/AgCl as the reference electrode. A 300-W Xe lamp served as a light source, and 0.5 M Na_2_SO_4_ solution was used as the electrolyte.

## Results and discussion

### Effects of hydrothermal reaction conditions

#### *Amount of EDTA*

**Figure 1:**
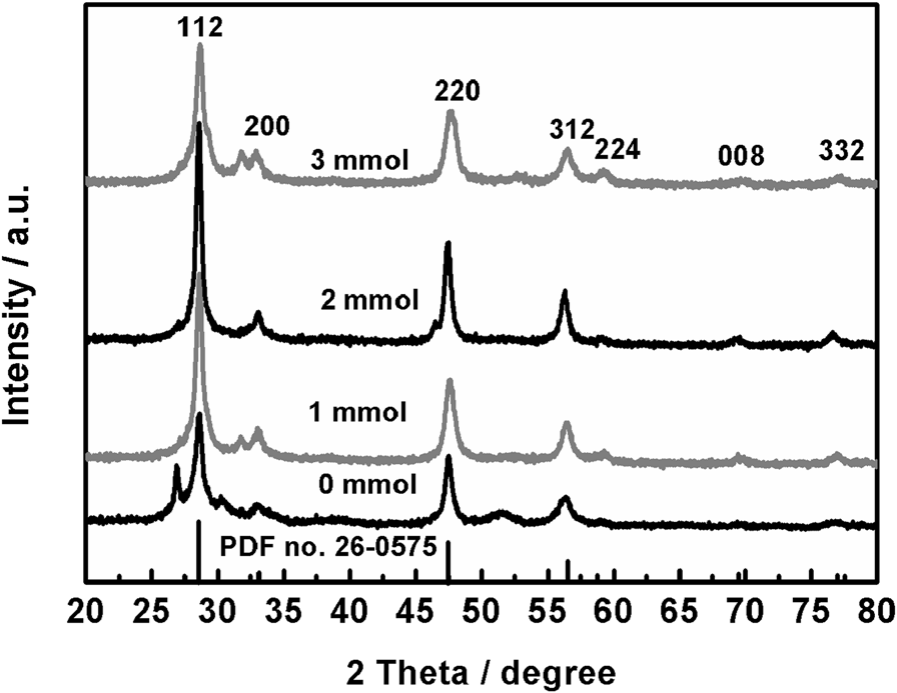
XRD patterns of the samples obtained under different amounts of EDTA.

In order to investigate the amount of EDTA on the phase composition of the obtained product, several samples have been synthesized at 180°C for 16 h by adding different amounts of EDTA into the reaction system with Cu/Zn/Sn at 2:2:1. Figure 1 displays the PXRD patterns of the samples. The sample obtained from the reaction system containing no EDTA shows seven diffraction peaks located at 26.8°, 28.7°, 30.3°, 33.0°, 47.6°, 51.4°, and 56.4°. According to the standard PXRD pattern of kesterite CZTS (PDF no. 26-0575), the four diffraction peaks located at 28.7°, 33.0°, 47.6°, and 56.4° can be attributed to (112), (200), (220), and (312) planes of kesterite CZTS, respectively. Note that a new wurtzite phase of CZTS was discovered by Lu et al. [8] and that the arrangements of atoms in the simulated wurtzite were basically similar to those in kesterite [34]. Consequently, the three strongest peaks located at 28.7°, 47.6°, and 56.4° can be also ascribed to (002), (110), and (112) planes of wurtzite CZTS, respectively. Besides, the diffraction peaks located at 26.8°, 30.3°, and 51.4° can be attributed to (100), (101), and (103) planes of wurtzite CZTS, respectively. It is revealed that the CZTS sample prepared from the reaction system containing no EDTA is a mixture of kesterite and wurtzite. The presence of the diffraction peak located at 33.0°, originated from (200) planes of kesterite CZTS, along with the absence of the diffraction peak located at around 39°, corresponding to (102) planes of wurtzite CZTS, implies that the content of kesterite is more than that of wurtzite in the CZTS sample. After 1 mmol of EDTA has been added into the reaction system, the obtained sample exhibits four main diffraction peaks of kesterite CZTS, together with one weak impurity peak located at 31.6°, which probably originates from CuS or Sn_2_S_3_. The absence of the diffraction peaks of wurtzite CZTS suggests that the addition of EDTA in the hydrothermal reaction system hampers the formation of wurtzite, thus favoring the production of pure kesterite CZTS. Furthermore, the PXRD pattern of the sample produced from the reaction system containing 2 mmol of EDTA is identical to the standard one of kesterite CZTS. The relatively high intensity of the diffraction peaks implies that the obtained sample is in high purity and good crystallinity. However, as the amount of EDTA is further increased to 3 mmol, the obtained sample exhibits the diffraction peaks of kesterite CZTS, together with one weak impurity peak located at 31.6°. The above results suggest that a suitable amount of EDTA added into the reaction system is essential for producing pure kesterite CZTS by the hydrothermal process. For the solvothermal process with *N*,*N*-dimethylformamide (DMF) as the solvent, EDTA was not needed for preparing pure kesterite CZTS, even if l-cysteine was also used as the sulfur source [30]. The reason for this difference is possibly due to the fact that the complex reactions between the three metal ions with l-cysteine take place more easily in DMF than in water. As a result, EDTA is needed for the process with water as the solvent and l-cysteine as the sulfur source, which is used as a complexing agent. It is obvious that the hydrothermal process provides an environment-friendly and low-cost route for producing pure kesterite CZTS, as compared with the solvothermal method with DMF as the solvent.

#### *Mole ratio of three metal ions*

**Figure 2:**
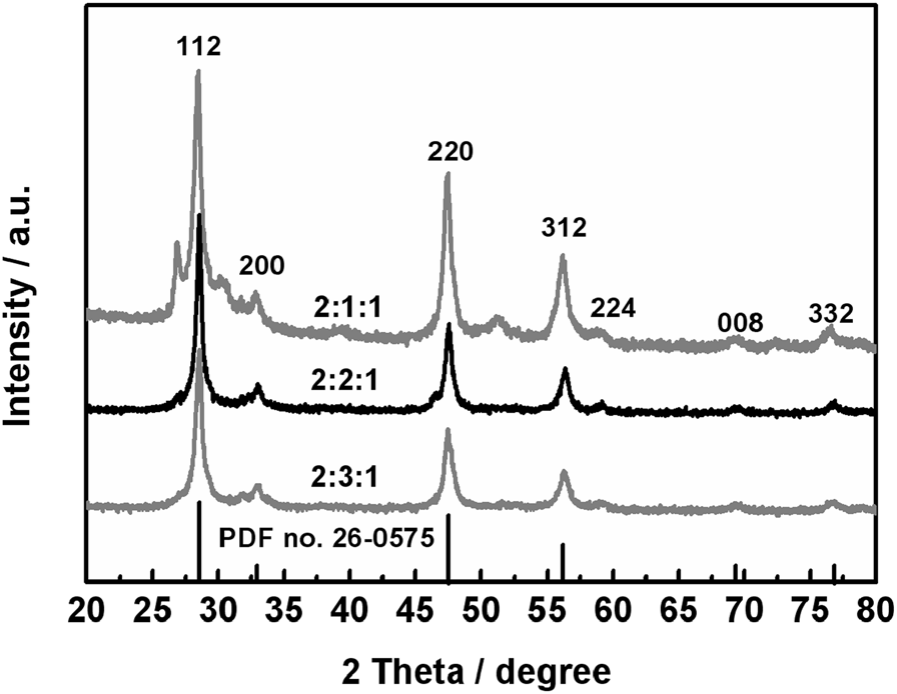
XRD patterns of the samples obtained at different Cu/Zn/Sn/S mole ratios.

The stoichiometric control of quaternary compounds is complicated by the tendency of forming a plurality of compositional phases, due to the difference in reactivity of the cationic precursors. Consequently, the mole ratio of the three cationic precursors in the reaction system should have an important effect on the phase composition of the obtained samples. Figure 2 shows the PXRD patterns of the samples synthesized at 180°C for 16 h from the reaction system containing 2 mmol of EDTA at different mole ratios of the three metal ions. At Cu/Zn/Sn = 2:1:1, corresponding to the stoichiometric ratio of CZTS, the obtained sample shows a similar XRD pattern to the one prepared from the reaction system containing no EDTA (Figure 1), implying that it has a mixed phase of kesterite and wurtzite. Besides, a weak impurity peak located at 31.7° appears. As the amount of ZnCl_2_ in the reaction system is doubled, and thus Cu/Zn/Sn is accordingly changed from 2:1:1 to 2:2:1, the obtained sample can be identified as kesterite CZTS in high purity and good crystallinity. Note that at Cu/Zn/Sn = 2:3:1, the obtained sample exhibits several diffraction peaks of kesterite CZTS, together with one weak impurity peak located at 31.8°. These results indicate that the mole ratio of the three cationic precursors influences the phase composition of the obtained product. An excessive dose of ZnCl_2_ (double the stoichiometric ratio of Zn in CZTS) in the reaction system favors the production of pure kesterite CZTS.

#### *Effect of hydrothermal temperature*

**Figure 3:**
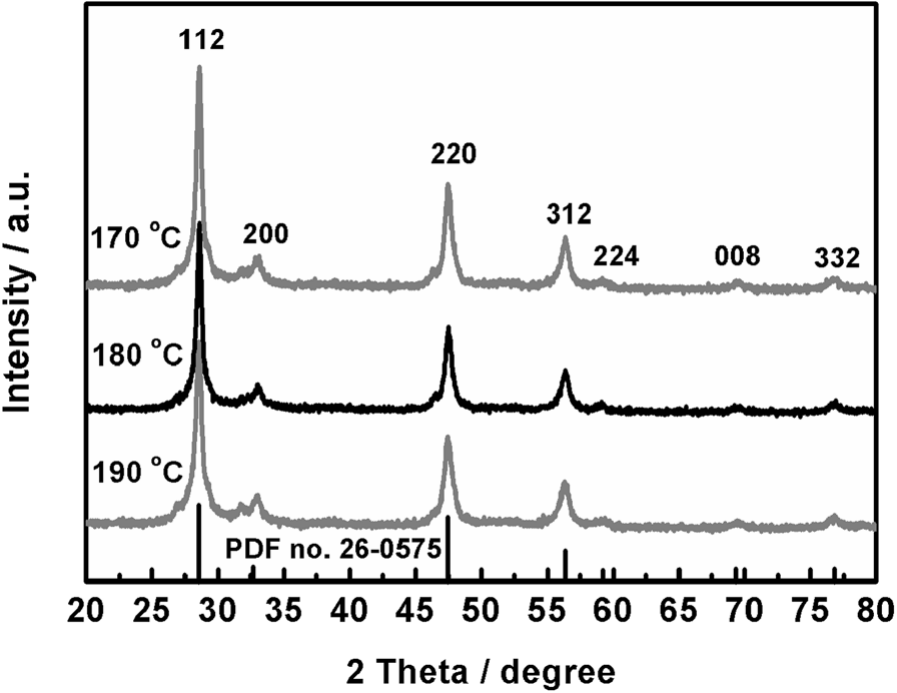
XRD patterns of the samples obtained at different hydrothermal temperatures.

With the amount of EDTA fixed at 2 mmol and Cu/Zn/Sn set at 2:2:1, the hydrothermal synthesis was conducted at different temperatures for 16 h. Figure 3 displays the PXRD patterns of the samples prepared at 170°C, 180°C, and 190°C. All the obtained samples show the seven diffraction peaks located 28.7°, 33.0°, 47.6°, 56.4°, 59.2°, 69.5°, and 76.7°, which are ascribed to (112), (200), (220), (312), (224), (008), and (332) planes of kesterite CZTS, respectively. However, the two samples prepared at 170°C and 190°C exhibit one weak impurity peak located at 31.8°. It is suggested that kesterite CZTS can be synthesized at the hydrothermal temperatures ranging between 170°C and 190°C from the reaction system containing 2 mmol of EDTA at 2:2:1 of Cu/Zn/Sn. The suitable temperature for producing pure kesterite CZTS should be around 180°C.

#### *Effect of reaction time*

**Figure 4:**
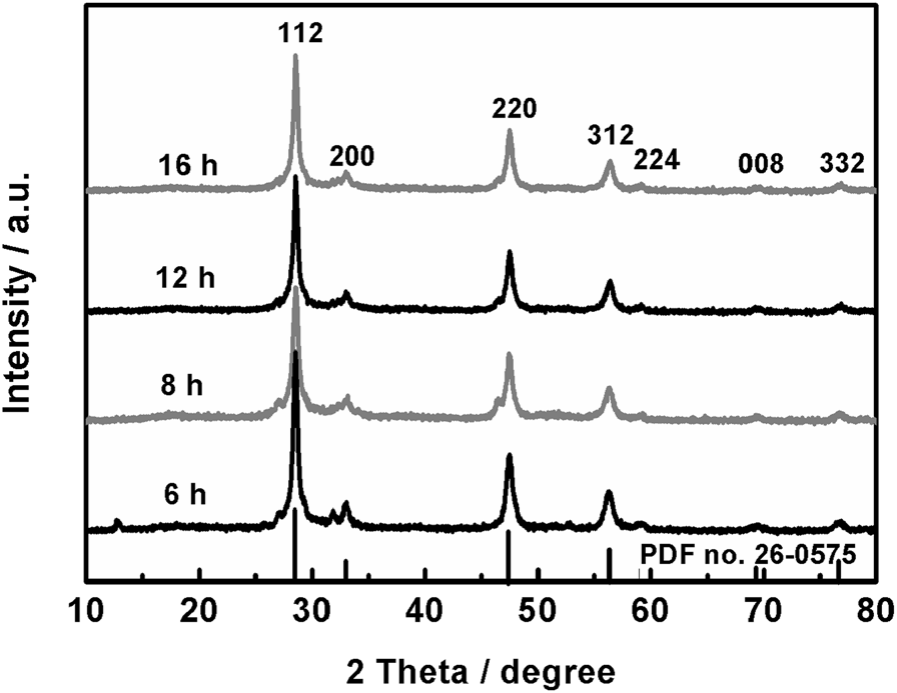
XRD patterns of the samples obtained at 180°C for different times.

In order to investigate the effect of the reaction time on the phase composition of the obtained product, four samples have been synthesized at 180°C for 16, 12, 8, and 6 h, respectively, and their PXRD patterns are displayed in Figure 4. As the reaction time is reduced from 16 to 12 h, the obtained sample still has the phase of kesterite in high purity and good crystallinity. However, as the reaction time is further reduced to 8 and 6 h, the obtained two samples show the weak impurity peaks located at 46.5° and 31.8°, respectively. These results imply that pure kesterite CZTS can be produced by the hydrothermal process at 180°C for no less than 12 h.

### Microstructure, morphology, and optical absorption property

**Figure 5:**
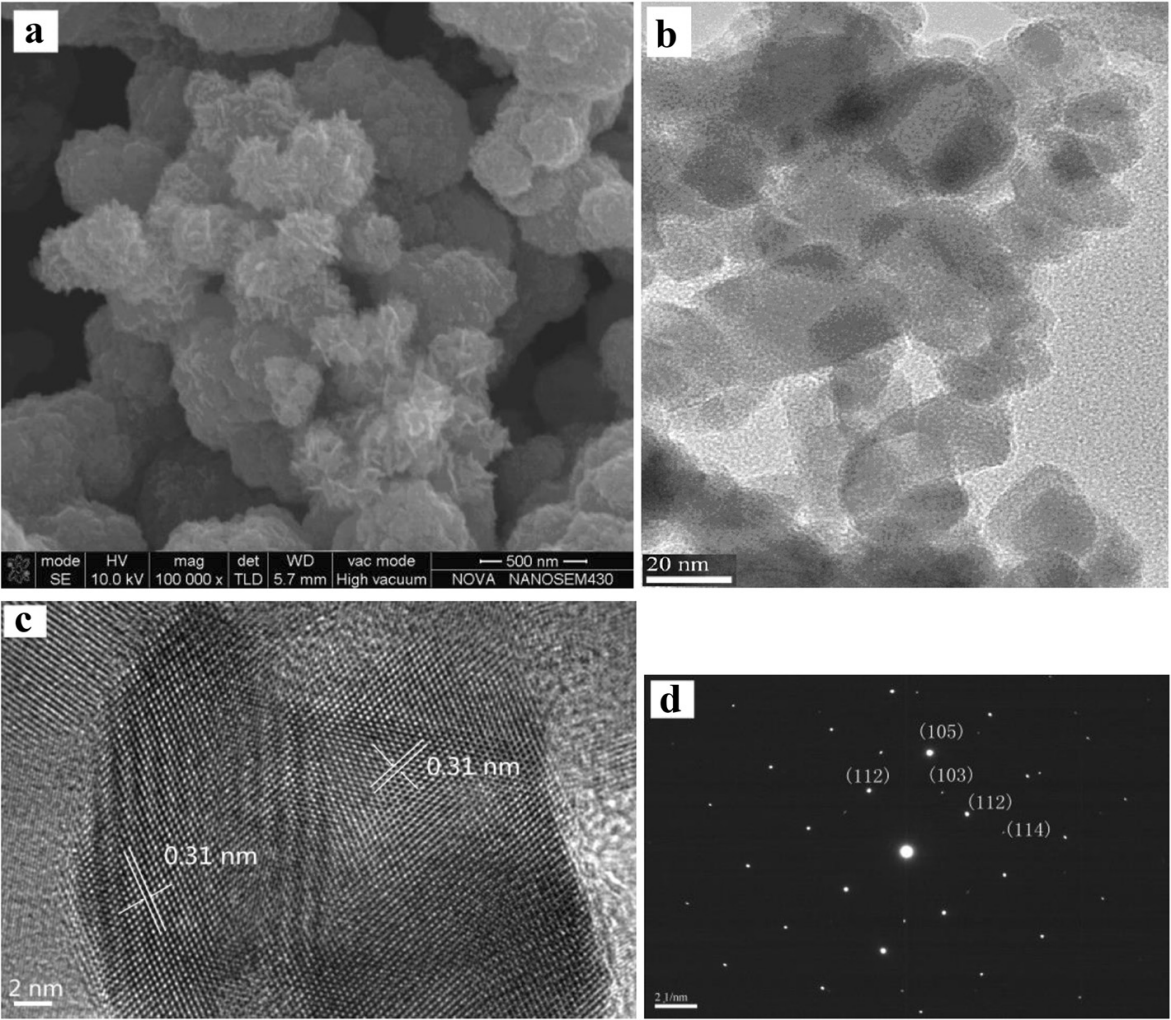
**SEM, TEM, and HRTEM images and SAED pattern of the CZTS sample prepared by hydrothermal method. (a)** SEM, **(b)** TEM, **(c)** HRTEM, and **(d)** SAED pattern.

Figure 5 shows SEM, TEM, and HRTEM images and a SAED pattern of the pure CZTS sample synthesized at 180°C for 12 h from the reaction system containing 2 mmol of EDTA at 2:2:1 of Cu/Zn/Sn. The SEM image (Figure 5a) reveals general morphologies of flower-like particles, which are assembled from nanoflakes. The sizes of the hierarchical particles range from 250 to 400 nm, much smaller than the microspheres (approximately 2.2 μm) prepared by the solvothermal method at 250°C for 8 h [18]. The observations of the CZTS sample by TEM and HRTEM were performed after it had been dispersed into ethanol by ultrasound. The TEM image (Figure 5b) shows some hexagonal nanoflakes with *ca.* 20 nm in size, implying that the hierarchical CZTS particles have been disassembled into the nanoflakes by ultrasound. As shown from the HRTEM image (Figure 5c), the continuous lattice fringes throughout a particle indicate the single crystalline nature of the nanoscale flakes, which is further confirmed by the dotted SAED pattern recorded for a single particle (Figure 5d). The *d*-spacing value has been calculated to be 0.31 nm (Figure 5c), identical to the theoretical value of 0.31 nm for (112) planes of kesterite CZTS.

**Figure 6:**
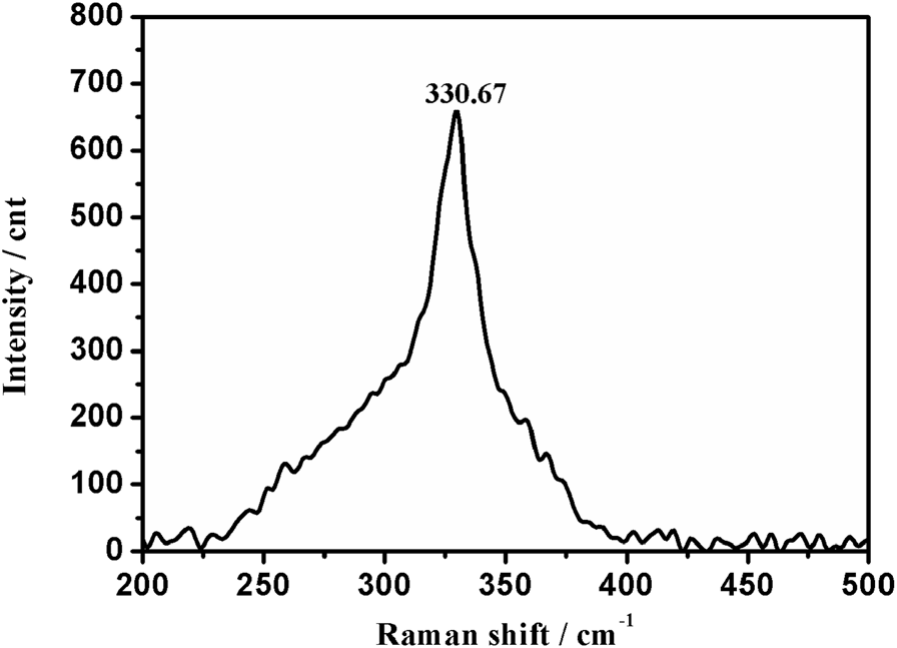
The room-temperature Raman spectrum of the hierarchical CZTS flower-like particles.

Some binary and ternary compounds including ZnS, Cu_3_SnS_4_, and Cu_2_SnS_3_ could be present as impurity in CZTS [35], and their PXRD patterns are similar to that of kesterite CZTS. As a result, it is hard to distinguish CZTS from those binary and ternary compounds by using XRD. In order to further confirm the phase composition of the hierarchical CZTS particles, room-temperature Raman spectroscopy has been employed due to the ability of this technique to distinguish between the CZTS phase and the ZnS, Cu_3_SnS_4_, and Cu_2_SnS_3_ phases. Figure 6 shows the room-temperature Raman spectrum of the hierarchical CZTS particles. The kesterite CZTS sample exhibits a high intensity peak at 330.67 cm^-1^, close to the reported Raman intense peak positions in the range from 331 to 338 cm^-1^ for kesterite CZTS and totally different from the characteristic peaks of the ZnS (351 cm^-1^), Cu_3_SnS_4_ (318 cm^-1^), and Cu_2_SnS_3_ (298 cm^-1^) phases. It can be inferred that those impurity phases are absent in the kesterite CZTS sample.

**Figure 7:**
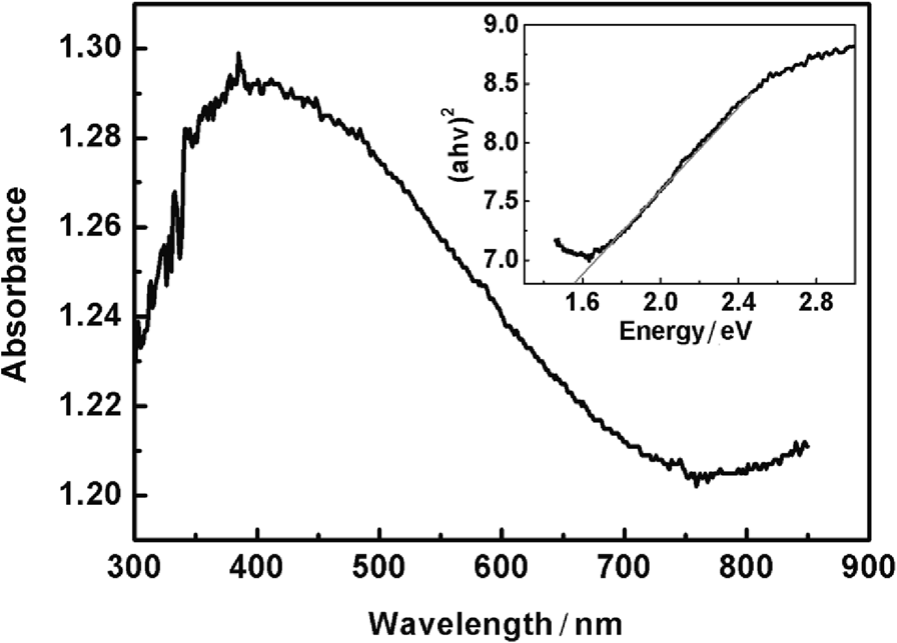
The UV-vis diffuse reflectance spectrum of the hierarchical CZTS flower-like particles.

Figure 7 shows the optical absorption spectrum obtained from diffuse reflectance of the hierarchical CZTS particles. The direct optical band gap of the CZTS particles has been calculated from the UV-vis spectrum to be 1.55 eV by extrapolation of the linear region of a plot of (*αhν*)^2^ versus energy (the inset in Figure 7), where *α* represents the absorption coefficient and *hν* is the photon energy. Compared to 1.48 eV of bulk CZTS, a blueshift of 0.07 eV in the band gap is observed for the hierarchical CZTS particles, which could be attributed to the quantum confinement effect originated from the CZTS single-crystal nanoflakes.

### Photoelectrochemical property of CZTS films

**Figure 8:**
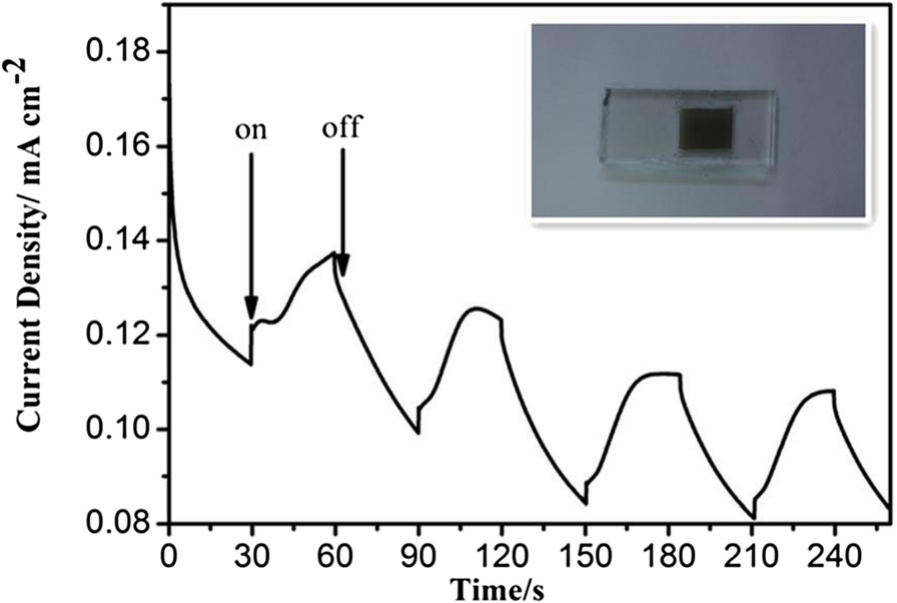
**The transient photocurrent responses (*I*-*t*) of the CZTS film at 0.5 V vs. Ag/AgCl.**

The hierarchical CZTS particles have been employed to fabricate films, and the photoelectrochemical property of the obtained CZTS films has been evaluated by measuring their transient current response (*I-t*) with several on-off cycles. Figure 8 shows the photoelectrochemical *I*-*t* curve of the CZTS film under intermittent visible-light irradiation (>420 nm) at 0.5 V vs Ag/AgCl, and a typical photograph of the film is inserted in this figure. The CZTS film exhibits fast photocurrent responses, indicating its good photoelectrochemical property. It can be suggested that the hierarchical CZTS particles synthesized by the facile and nontoxic hydrothermal route show potentials for use in solar cells and photocatalysis.

## Conclusions

The reaction conditions including the amount of EDTA, the mole ratio of the three metal ions, and the hydrothermal temperature and time have an important effect on the phase composition of the obtained product. A suitable amount of EDTA is needed for synthesis of pure kesterite CZTS by the hydrothermal process with l-cysteine as the sulfur source. An excessive dose of ZnCl_2_ (double the stoichiometric ratio of Zn in CZTS) in the reaction system favors the production of kesterite CZTS. Pure kesterite CZTS can be produced by the hydrothermal process at 180°C for no less than 12 h. It is confirmed that those binary and ternary phases are absent in the kesterite CZTS product. The kesterite CZTS material synthesized by the hydrothermal process consists of flower-like particles with 250 to 400 nm in size. The particles are assembled from the single-crystal CZTS nanoflakes with *ca.* 20 nm in size. The band gap of the CZTS material is estimated to be 1.55 eV. The CZTS films fabricated from the flower-like CZTS particles exhibit fast photocurrent responses, making them show potentials for use in solar cells and photocatalysis.

## Competing interests

The authors declare that they have no competing interests.

## Authors' contributions

YX designed and conducted the experiments, carried out the experimental analyses, and drafted the manuscript. ZC fabricated the films and performed the photoelectrochemical measurement. ZZ, XF, and GL conceived the study, participated in its design and coordination, wrote the introduction, and modified the manuscript. All authors read and approved the final manuscript.
